# The gut microbiome as a target for regulatory T cell-based immunotherapy: induction of regulatory lymphocytes by oral administration of anti-LPS enriched colostrum alleviates immune mediated colitis

**DOI:** 10.1186/s12876-015-0388-x

**Published:** 2015-10-30

**Authors:** Ami Ben Yaʼacov, Yoav Lichtenstein, Lidya Zolotarov, Yaron Ilan

**Affiliations:** Gastroenterology and liver Unit, Department of Medicine, Hadassah Hebrew University Medical Center, Jerusalem, Israel

**Keywords:** Microbiome, Regulatory T cells, Colostrum, Inflammatory bowel disease, Crohn’s disease

## Abstract

**Background:**

Gut-derived bacterial endotoxin is an important cofactor in the pathogenesis of IBD. Regulatory T cells (Tregs) are essential for maintenance of peripheral tolerance and can prevent and alleviate IBD. To determine the immune modulatory effect of anti-LPS enriched hyperimmune colostrum, its ability to induce Tregs and alleviate immune mediated colitis.

**Methods:**

Immune-mediated colitis was induced in mice by intra-colonic instillation of Trinitrobenzene Sulfonate (TNBS). Four groups of mice were orally administered with two dosages of IgG-enriched colostrum fractions. The fractions were harvested from cows immunized against LPS derived from intestinal *Escherichia coli* bacteria (Imm124E). Control mice received non-immunized colostrum or vehicle (PBS). Treatment was administered one day following sensitization and four additional days following the administration of TNBS. The following parameters in the mice were tracked: body weight, bowel histology, serum cytokine levels and regulatory T cells.

**Results:**

Oral administration of Imm124E hyperimmune colostrum ameliorated immune-mediated colitis. Significant amelioration of weight reduction was noted in treated mice. Oral administration of Imm124E improved bowel histology. Both the extent of the disease, inflammation score, and colitis damage and regeneration scores decreased in Imm-124E treated animals. These effects were associated with an increase in serum IL10 anti inflammatory cytokine levels, and an increase in CD4 + CD25+ and CD4 + Foxp3+ Tregs.

**Conclusions:**

Oral administration of Imm124E promoted Tregs and alleviated bowel inflammation in immune mediated colitis. The present data suggests that the microbiome may serve as a target for Tregs-based immunotherapy.

**Electronic supplementary material:**

The online version of this article (doi:10.1186/s12876-015-0388-x) contains supplementary material, which is available to authorized users.

## Background

The human gut is a natural environment for a dynamic microbial ecosystem associated with immune mediated disorders [1]. Several disease states have been associated with changes in the composition of fecal and intestinal mucosal communities, including inflammatory bowel diseases (IBD), obesity and metabolic syndrome [1]. Chronic inflammation involves endotoxins derived from the gut flora.

Endogenous gut-derived bacterial endotoxin has been considered an important cofactor that mediates the pathogenesis of IBD [2]. Aberrant immune responses toward commensal gut bacteria result in the onset and perpetuation of IBD [3]. Reduced microbiotic diversity in conjunction with a lower proportion of Gram positive and higher proportion of Gram negative bacteria compared with that of healthy subjects is reported in IBD patients [3]. In a subset of IBD patients, E. coli strains with specific features trigger activation of the disease. Antibiotics can decrease tissue invasion and eliminate aggressive bacterial species [2]. They are used in IBD to treat infective complications and also for altering bacterial flora, which may result in specific anti-inflammatory effects. Therapeutic manipulation of the intestinal flora offers considerable promise for treating IBD [2].

Regulatory T cells (Tregs) have been demonstrated to be essential for maintaining peripheral tolerance, preventing autoimmune diseases and limiting chronic inflammatory diseases [4]. Induction of mucosal tolerance is effective in the treatment of autoimmune and inflammatory diseases due to the lack of toxicity, ease of administration and antigen-specific mechanism of action [5]. Oral administration of anti-CD3 antibodies induces Tregs through a TGF-β-dependent mechanism, alleviating immune mediated disorders including colitis.[6–9].

Bovine colostrum (BC) contains abundant bioactive components including growth factors, immunoglobulin (Igs), lactoperoxidase, lysozyme, lactoferrin, nucleosides, vitamins, peptides and oligosaccharides [10, 11]. Colostrum is also rich in cytokines and other immune agents that provide bacteriostatic, bactericidal, antiviral, anti-inflammatory and immunomodulatory protection against infection [12, 13]. Hyperimmune colostrum raised against a bacterial LPS extract (Imm124-E, Immuron Ltd, Melbourne, Australia) was shown beneficial in animal models of immune-associated insulin resistance and non-alcoholic steatohepatitis [14]. These beneficial effects were associated with an increase in the number of splenic CD4 + CD25+, CD4 + CD25 + Foxp3+ and CD3 + NK1.1+ regulatory lymphocytes. In humans with metabolic syndrome, oral administration of Imm124-E was safe and alleviated insulin resistance, liver damage, and hyperlipidemia [15]. These effects were accompanied by increased serum levels of glucagon-like peptide 1 (GLP-1), adiponectin and Tregs.

The aim of the current study was to determine in experimental TNBS-induced colitis the effects of hyperimmune colostrum preparations enriched with IgG-enhanced colostrum fractions. These colostrum preparations were harvested from cows immunized against LPS from intestinal bacteria (*Escherichia coli,* Imm124E). Our results indicate that oral administration of Imm124E promoted Tregs and alleviated the inflammatory state in this model.

## Methods

### Colostrum collection and processing

Colostrum preparations were prepared and provided by Immuron Ltd. (Melbourne, Australia). Colostrum was collected from immunized or non-immunized cows (colostrum control) and was frozen in individual bags. Each batch was subsequently pasteurized and concentrated by ultra-filtration before freeze-drying. Colostrum preparations were manufactured and tested by an accredited testing lab (Dairy Technical Services, Melbourne, Australia) with regard to specifications for the levels of IgG protein, moisture, lactose, fat, antibiotics and microbiology parameters. All first-milking colostrum powder preparations contained approximately 40 % IgG (w/w). All freeze-dried bovine colostrum powders were emulsified in water and stored at 4 °C prior to administration to mice.

### E. coli antigens

Killed bacterial antigens from the single ETEC strain (O78) vaccine or from the multiple ETEC strain vaccine (O6, O8, O15, O25, O27, O63, O114, O115, O128, O148, O153 and O159 serotypes) were used as a source of antigen for coating ELISA plates. Stock antigens were prepared according to the manufacturer’s instructions (Allied Biotechnology, Australia). LPS from Enterotoxigenic E. coli (ETEC) colostrum was designated as Imm124-E. IgG purified from Imm124-E was prepared using a Prosep G column to purify colostrum powder [14]. An in house ELISA test was used to assess the levels of immunoglobulin in the powder as described [14].

### Animals and experimental design

Male, Balb/c, 7–8 week old mice were purchased from Harlan (Jerusalem, Israel). Mice were maintained in the Animal Core of the Hadassah-Hebrew University Medical School. All mice were administered standard laboratory chow and water ad libitum and maintained under a 12 h light/dark cycle.

### Induction of colitis

To induce TNBS- colitis, mice were exposed by skin painting with 160 μL of the haptenizing agent TNBS (Sigma-Aldrich, Rehovot, Israel) at a concentration of 2.5 % in 50 % ethanol on day −7. On day 0, 120 μL of 2.5 % TNBS in 50 % ethanol was administered intrarectally via a 3.5-French catheter which was carefully inserted into the colon ensuring that the tip was 4 cm proximal to the anus. Four groups of mice (4 mice per group) were orally administered either vehicle (PBS); 100 μg of colostrum control; 50 μg of Imm124-E, or 500 μg of Imm124-E daily, starting one day following intrarectal administration of TNBS and for four additional days following administration of TNBS, and then sacrificed.

### Follow up of the effects of Imm124E on immune mediated colitis

- Effect on body weight:. Mice were weighed on day −7 (beginning of experiment), on day 0 (intrarectal TNBS) and on the sacrifice day.
- Grading of colon histology: For histological evaluation of the inflammation, distal colonic tissue (last 10 cm) was removed after the mice were sacrificed, and then fixed in 10 % formaldehyde. Paraffin sections from each animal were then stained with hematoxylin- eosin according to standard techniques. The degree of inflammation, its extent, damage and regeneration seen on microscopic cross sections of the colon was graded from 0 to 3 with grade 0 being considered normal, as described [16].
- Effect of cytokine serum levels: Serum levels of IL-10 and IFNγ were determined by “sandwich” ELISA using a commercial kit according to the manufacturer’s recommended instructions (Quantikine, R&D Systems, Minneapolis, MN, USA).
- Effect on Regulatory T cells: Flow cytometry was performed following splenocyte and intrahepatic lymphocyte isolation using 1x10^6^ lymphocytes in 100 ml PBS with 0.1 % BSA. For surface staining, cells were incubated with fluorochrome-conjugated antibodies to the indicated cell-surface markers (eBioscience, San Diego, CA, USA) at the recommended dilution or with isotype control antibodies for 30 min at 4 °C. The following cell surface anti-mouse antibodies were used: CD4-eFluoro 450, CD8- FITC and CD25-PE. Cells were then washed in PBS containing 1 % BSA and fixed with fixation buffer (eBioscience) for another 50 min. For intracellular staining of Foxp3, cells after fixation were permeabilized with Foxp3 staining buffer (eBioscience). The resulting cells were stained with PE-Cy7-conjugated antibodies to Foxp3 (eBiosciences). Cells were then washed twice and re-suspended in 250 μl of PBS containing 1 % BSA and kept at 4oC. 1x10^6^ stained cells in 250 μl of PBS containing 1 % BSA were subsequently analyzed using a FACS LSR II instrument (Becton Dickinson, San Jose, CA) with FCS express V.3 software (DeNovo software, CA, USA). Only live cells were counted, and background fluorescence from non-antibody-treated lymphocytes was subtracted.

### Statistical analyses

Comparison of the two independent groups was performed using Student’s *t*-test. All tests that we applied were two-tailed, and a *p* value of 0.05 or less was considered statistically significant.

## Results

### Oral administration of Imm-124E ameliorated the weight loss in immune mediated colitis

**Fig. 1:**
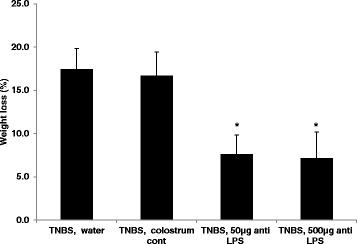
Following the induction of TNBS colitis, mice were monitored for body weight. Oral administration of Imm-124E ameliorated weight reduction compared with the control group that received PBS or non-immune colostrum

Figure 1 shows that oral administration of Imm-124E was associated with significant weight loss amelioration. Mice in control groups that received PBS or colostrum control lost weight (17 % and 16.8 % decrease from baseline, respectively). In contrast, in the mice treated with the low and high doses of immunized colostrum, Imm-124E, we noted a milder decrease in weight (7.5 % and 7.1 %, decrease, respectively, *P* = 0.001 and *P* = 0.002, respectively).

### Oral administration of Imm-124E ameliorated the colonic damage in immune mediated colitis

**Fig. 2:**
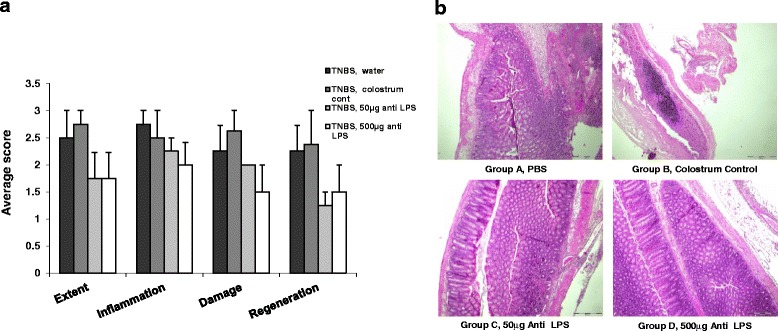
The effect of oral administration of Imm-124E on colonic damage in immune mediated colitis. **a**. Histologic scores were performed on bowel biopsies taken from all mice in all treated and control groups. Sections were scored for extent of disease, degree of inflammation, damage and regeneration. **b**. Representative sections from each of the groups are shown (X10). Animals in the control groups showed significant inflammation affecting all layers of the colon while almost normal mucosal sections were noted in both the low and high dosages of Imm-124E treated mice

Figure 2a shows that oral Imm-124E improved colon histology. The extent of disease decreased from 2.5 and 2.75 in PBS and colostrum control treated mice, compared with 1.75 and 1.75 in the low dose and high dose of Imm-124E -treated animals, respectively. Similarly, the inflammation score decreased from 2.75 and 2.5 in PBS and non-immune colostrum treated mice to 2.25 and 2.0 in the low dose and high dose Imm-124E treated mice, respectively; The damage score decreased from 2.25 and 2.65 in PBS and non-immune colostrum treated mice, to 2.0 and 1.5 in the low dose and high dose Imm-124E treated animals, respectively; The regeneration score decreased from 2.25 and 2.75 in PBS and non-immune colostrum treated mice, to 1.25 and 1.5 in the low dose and high dose Imm-124E treated animals, respectively (*P* = NS).

Figure 2b shows representative colon sections from each of the groups. While animals in the control groups showed signs of inflammation affecting all layers of the colon mucosa, almost normal mucosal sections were noted in both the low and high dosage Imm-124E treated mice.

### Oral administration of Imm-124E increased IL10 serum levels in immune mediated colitis

**Fig. 3:**
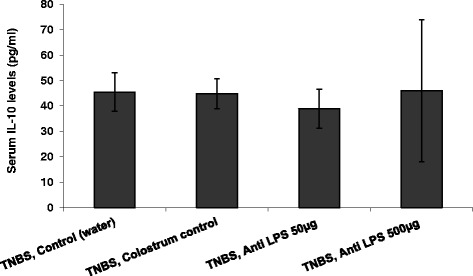
The effect of oral administration of high dose Imm-124E on IL10 serum levels as measured by ELISA

Figure 3 shows that oral administration of high dose Imm-124E was associated with an increase in serum levels of IL10 (*p* < 0.005 for mice treated with 500 microgram vs. controls). No effect was noted on IFNγ serum levels.

### Oral administration of Imm-124E promoted regulatory T cells

**Fig. 4:**
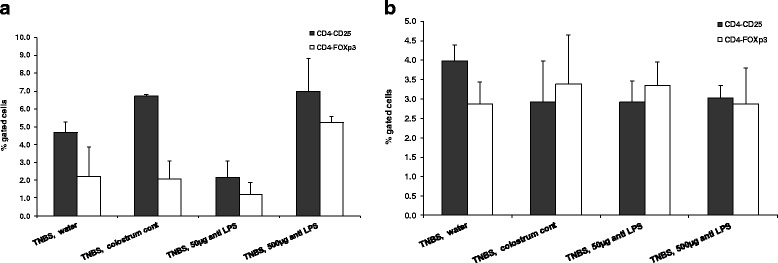
FACS analysis was performed on lymphocytes harvested from spleens (4**a**) and livers (4**b**) of animals in all groups. A high dosage of Imm-124E led to an increase in both CD4+CD25+ and CD4+Foxp3+ intrasplenic lymphocyte subsets. No effect was noted on intrahepatic lymphocyte subsets

Figure 4a shows that the high dosage of Imm-124E promoted both CD4 + CD25+ and CD4 + Foxp3+ intrasplenic lymphocyte subsets. An increase in CD4 + CD25+ subset was noted in Imm-124E treated mice and in mice treated with colostrums, compared with controls (*P* = NS). An increase was noted for CD4 + Foxp3+ in mice treated with the high dose Imm-124E (*P* = NS). No effect was noted on the intrahepatic lymphocyte subsets (Fig. 4b).

## Discussion

The results of the present study show that oral administration of hyperimmune colostrum preparations enriched with anti-LPS antibodies that are harvested from cows immunized against LPS from intestinal bacteria (*Escherichia coli*), ameliorated TNBS-induced colitis. Oral administration of Imm-124E ameliorated weight loss that was associated with improved disease clinical course and colon histology. The extent of disease, inflammation score, and colitis damage and regeneration scores decreased in Imm-124E- treated animals. These effects were associated with increased serum IL10 levels and a trend towards increased levels of Tregs (CD4 + CD25+ and CD4 + Foxp3+ subsets).

The gastrointestinal tract contains a large and diverse population of commensal bacteria and is also one of the primary sites of exposure to pathogens. Intestinal microbes play a role in both the immune system and in maintaining metabolic homeostasis [17]. In a healthy host the immune system is tolerant to gut bacteria and does not mount an effector response to bacteria-derived antigens [18]. Thus, the intestinal immune system is instructed by the microbes to limit responses to luminal antigens [17]. Gut microbes can inhibit the transport of both commensal and pathogenic bacteria from the lumen to the mesenteric lymph nodes (MLNs) [17].

Breakdown of tolerance at the gut level may contribute to the development of IBD [19]. This breakdown may be associated with bowel infection. The compartmentalization of systemic and gut mucosal immunity apparatuses focuses adaptive immunity on gut microbes specifically at the level of the bowel. In circumstances of increased gut microbial exposure due to elevated gut epithelial permeability resulting from genetic deficiencies in local defense mechanisms or during gastrointestinal pathogenic infections, this paradigm is broken and a systemic immune response towards gut microbes is induced [20]. The loss of tolerance to commensals during bowel infections leads to activation of microbiome-specific T cells which differentiate into inflammatory effector cells [19]. These T cells may go on to form memory cells that are pathogen-specific. The response to commensals during infection in the bowel parallels the immune response to pathogenic microbes, suggesting that the adaptive responses against commensals are an integral component of mucosal immunity [19]. In the absence of Myd88 or with antibiotic-induced dysbiosis, non-invasive bacteria were trafficked to MLNs in a CCR7-dependent manner, inducing an inflammatory response [17].

The data of the present study supports a possible correlation between specific E. Coli strains and IBD. Following enterocyte stress, translocation of E. coli strains C25 and HBTEC-1 across Caco-2 monolayers was markedly stimulated and accompanied by an increase in internalization into the enterocytes. The data suggested that E. coli strains capable of translocation may comprise a separate group of E. coli strains [21]. Enteropathogenic (EPEC) and enterohaemorrhagic (EHEC) strains of E. coli use a type III secretion system (T3SS) to deliver virulence effector proteins into host cells during infection. This system enables bacterial colonization while interfering with the antimicrobial host response [22]. In a recent study it was shown that a T3SS effector NleB1 from EPEC binds to host cell death-domain-containing proteins and thereby inhibits death receptor signaling [22]. NleB1 prevented Fas ligand or TNF-induced formation of the canonical death-inducing signaling complex (DISC) and proteolytic activation of caspase-8, an essential step in death-receptor-induced apoptosis. NleB activity suggests that EPEC antagonize death-receptor-induced apoptosis of infected cells, thereby blocking a major antimicrobial host response [22]. These findings may be of importance for a possible association between specific strains of E. Coli and IBD. Increased gut permeability caused by the disruption of intracellular tight junctions in the intestine correlates with a high prevalence of small intestine bacterial overgrowth [23, 24]. Translocation of normally non-pathogenic bacteria across the gut may be a driving force of inflammatory responses associated with IBD [21]. Translocation may not be purely passive, but may occur via transcellular pathways activated in enterocytes by inflammatory or metabolic stress.

It was suggested that in human patients with a higher than average prevalence of small intestinal bacterial overgrowth there is an increased bacterial translocation [25]. Dysregulation of the intestinal immune response may increase the risk of exposure, something that may lead them to develop Crohn’s disease [25]. Serum lipopolysaccharide-binding protein (LBP) is a marker of disease activity in CD with a similar accuracy as that of the highly sensitive-CRP. LBP served as an independent predictor for 1-year clinical flare-up in these patients [26]. Studies have demonstrated that patients with Crohn’s disease have a shorter bowel length which may predispose them to the development of the disease via alterations of intestinal motility and changes in intestinal flora [25].

In the present study oral administration of hyperimmune colostrum preparations enriched with anti LPS effectively alleviated immune mediated colitis in mice and was associated with a significant induction of CD4 + CD25+ and CD4 + Foxp3+ regulatory T cells in the spleens of treated mice. Promotion of Tregs by oral administration of Imm124-E also improved insulin resistance, liver enzymes, hepatic fat accumulation and serum lipid profiles in mice and in humans [14, 15].

Tregs are critical for maintaining immune homeostasis and establishing tolerance to foreign, non-pathogenic antigens including those found in commensal bacteria and food [27]. Absence of Tregs or defective Treg function correlate with disease severity [28]. Tregs have suppressive mechanisms and control the threshold for peripheral antigen recognition via tonic down-regulation of dendritic cell (DC) co-stimulation. They are also implicated in maintaining the tolerogenic function of DCs [29]. Defects in Treg number or function lower the activation threshold, allowing proliferation and differentiation of self-reactive CD4 cells. Failure to maintain the tolerogenic commitment of DCs exposed to commensal microbes and allergens results in IBD [29]. Tregs were suggested to be beneficial in the context of IBD [27].

The human gut microbiome is important for interactions at both the intestinal level and the colonic epithelial cells level, with dendritic cells, and T and B immune cells [30]. These interactions affect gut barrier and defense mechanisms. Microbiotic composition may determine T effector- and Tregs balance and thus plays a role in the pathogenesis of IBD [30]. Alterations in the gut’s microbiome, acting via Tregs, were responsible for increased incidence of IBD [29]. Treg-based immunotherapy could potentially be custom-designed *ad hoc* to specifically suit each patient with limited side effects based on their individual microbial signatures [31].

A synergistic effect between anti-LPS antibodies and adjuvants in the colostrum is suggested by the results of the present study. This notion is based on the effect of the colostrums enriched with anti LPS antibodies compared to that of the control group that received antibody-free colosturm. Bovine colostrum (BC) treatment has been shown to be associated with an increased number of Tregs [15, 32, 33]. CD25 expression on peripheral blood monocytes (PBMCs) was enhanced by treatment with BC-derived IL-1β, TNF-β and IFN-γ [34]. Exposure to BC increased the percentage of cells expressing CD11a, CD11c, and CD43 and decreased the percentage of cells expressing CD62L, which facilitates lymphocyte trafficking [33, 35]. BC also contains defensin proteins, osteopontin, exosomes, TLRs, cathelicidin, eosinophil-derived neurotoxin LL-37 and high levels of β-glycosphingolipids (BGS) [15, 33, 36]. Some of these mediators serve as mucosal adjuvants, enhancing the interaction between subsets of antigen presenting cells and Tregs in the bowel mucosa [37, 38].

The present data suggests that oral antibodies have an active effect on the adaptive immune system. These results further support the possibility of altering the adaptive immune system by feeding of antibodies. Oral administration of anti-CD3 both in mice and humans altered the systemic immune response via presentation of antibodies at the gut level [6, 39, 40].

In summary, oral administration of Imm124E promoted Tregs and alleviated inflammation of the bowel in the immune-mediated colitis model. The data supports an association between gut bacterial translocation and Tregs, and suggests that the microbiome may serve as a target for Tregs-based immunotherapy.

## Conclusions

### Study strengths

Shows for the first time the effect of anti-LPS antibodies in animal model of colitis. It also emphasizes the synergistic effect between anti-LPS antibodies and the potent colosturm-derived immune adjuvants.

### Study limitations

Some of the parameters tested did not reach statistical significance and only trends are shown.

### Ethics

All animal experiments were performed according to the guidelines of the Hebrew University-Hadassah Institutional Committee for the Care and Use of Laboratory Animals and with the approval of the committee.

### Availability of data and materials

The data supporting their findings can be found as Additional file 1.

## Additional file

Additional file 1:
**Supplemenatry file with data of experiments.** (XLS 116 kb)

## Abbreviations

APCs: antigen presenting cells
BC: bovine colostrum
BC: bovine colostrum
BGS: β-glycosphingolipids
EHEC: enterohaemorrhagic E. Coli
EPEC: Enteropathogenic Escherichia coli
ETEC: Enterotoxigenic *E. coli*
GC: β-glucosylceramide
LBP: lipopolysaccharide-binding protein
MLNs: mesenteric lymph nodes
NKT: natural killer T cells
PBMCs: peripheral blood monocytes
T3SS: type III secretion system
TNF-α: Tumor Necrosis Factor-α
Tregs: regulatory T cells
